# The complete chloroplast genome of Russian sage *Salvia yangii* B. T. Drew (Lamiaceae)

**DOI:** 10.1080/23802359.2020.1781581

**Published:** 2020-06-24

**Authors:** Meng-Ting Cao, Jun-Jie Wu, Rui-Hong Wang, Ling Xu, Zhe-Chen Qi, Yu-Kun Wei

**Affiliations:** a Zhejiang Province Key Laboratory of Plant Secondary Metabolism and Regulation, College of Life Sciences and Medicine, Zhejiang Sci-Tech University, Hangzhou, China; b Shanghai Chenshan Plant Science Research Centre, Chinese Academy of Sciences, Shanghai Chenshan Botanical Garden, Shanghai, China

**Keywords:** *Salvia yangii*, *Perovskia atriplicifolia*, chloroplast genome, phylogenetics

## Abstract

The complete chloroplast genome of Russian sage *Salvia yangii* B. T. Drew was assembled in this study. The genome is 151,473 bp in length and contained 129 encoded genes in total, including 84 protein-coding genes, eight ribosomal RNA genes, and 37 transfer RNA genes. The result of phylogenetic analysis based on 15 chloroplast genomes revealed that *S. yangii* is closely related to common sage (*Salvia officinalis*) in Lamiaceae.

Russian sage (*Salvia yangii* B. T. Drew), previous *Perovskia atriplicifolia*, was recently included in the genus *Salvia* (Drew et al. 2017). It is a flowering herbaceous perennial plant and subshrub distributed in the steppes and hills of southwestern and central Asia. Due to its adaptability to soil and climate conditions, some cultivars, such as ‘Blue Spire’, have been widely used in gardening. Additionally, as a traditional medicinal plant in its native region, *S. yangii* is mainly used to treat rheumatism (Majetich and Zou 2008). Recent studies have shown some compounds such as abrotandiol, lariciresinol, syringaresinol, and taxiresinol extracted from this plant possess the antidiabetic and anti-HBV activity (Jiang et al. 2015; Butt et al. 2019). In this study, we assembled and characterized the first complete chloroplast genome of *S. yangii*, which will provide organelle molecular basis for further research of this horticultural and medicinally important plant.

The whole-genome shotgun sequencing data of *S. yangii* leave were retrieved from sequence read archive database of NCBI (Accession: SRR6940082). The leaves of *S. yangii* were provided by Mountain Valley Growers, Squaw Valley, CA (GPS: 36°40′48.0″N 119°10′02.7″W; Voucher: N. García 4533, deposited at University of Florida Herbarium). In total, about 4.8 G high-quality clean reads (150 bp PE read length) were obtained with adaptors trimmed. Following Liu et al. (2017, 2018), NOVOPlasty (Dierckxsens et al. 2017) was used to *de novo* assemble the chloroplast genome with *rbcL* gene as seed. Aligning and annotation were conducted using GeSeq and GENEIOUS prime (Biomatters Ltd, Auckland, New Zealand).

The complete chloroplast genome of *S. yangii* (GenBank accession No. MT537168) has a length of 151,473 bp with a typical circle structure and a GC content of 38.1%. It consists of a large single-copy region (LSC, 82,701 bp, 36.2% GC content), a small single-copy region (SSC, 17,566 bp, 31.9% GC content), and two inverted repeat regions (IR, 25603 bp, 43.1% GC content). In total, there were 129 genes in *S. yangii*, including 84 protein-coding genes, eight rRNA genes, and 37 tRNA genes. Among them, six protein-coding genes (*rpl2*, *rpl23*, *ycf2*, *ycf15*, *ndhB*, and *rps7*), seven tRNA genes (*trnI-CAU*, *trnL-CAA*, *trnV-GAC*, *trnI-GAU*, *trnA-UGC*, *trnR-ACG*, and *trnN-GUU*), and all four rRNA genes (*rrn16*, *rrn23*, *rrn4.5*, and *rrn5*) have two copies. Nine of these protein-coding genes (*rps16*, *atpF*, *rpoC1*, *petB*, *petD*, *rpl16*, *rpl2*, *ndhB*, and *ndhA*) have one intron each and two (*ycf3* and *clpP*) of them have two introns.

Sixteen species with available chloroplast genomes in Lamiaceae were selected to study the phylogentic placement of *S. yangii* (Figure 1). The sequence alignment was conducted by MAFFT v1.3 (Katoh and Standley 2013). We drew phylogenetic tree by the software IQtree (Nguyen et al. 2015) with 5000 bootstrap replicates and TVM + F+R2 model. The result of phylogenetics analysis showed that *S. yangii* is sister to *Salvia officinalis* in Lamiaceae.

**Figure 1.**
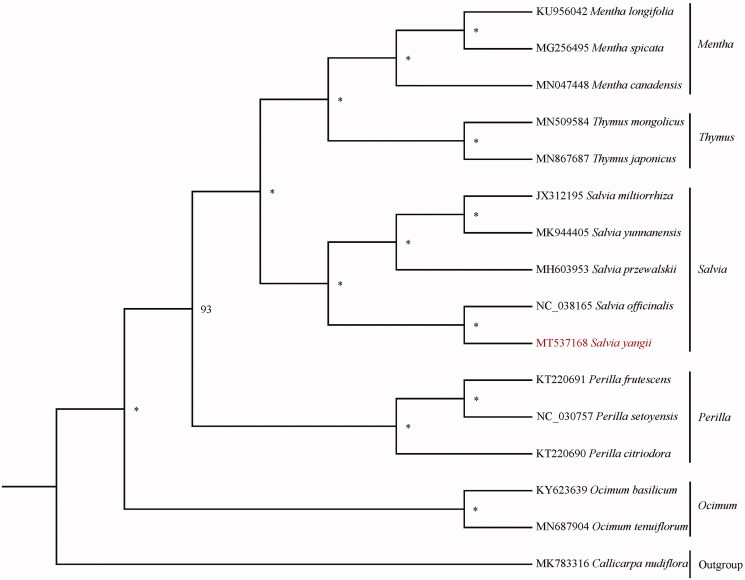
The phylogenetic tree based on 15 complete chloroplast genome sequences in Lamiaceae (accession numbers were listed in front of their names and ‘*’ indicates the bootstrap support values = 100).

## Data Availability

The DNA matrix and phylogenetic tree that support the findings of this study are openly available in Github at https://github.com/andresqi/Russian-sage-chloroplast-genome
